# Iron-Sulfur Cluster Complex Assembly in the Mitochondria of *Arabidopsis thaliana*

**DOI:** 10.3390/plants9091171

**Published:** 2020-09-09

**Authors:** Alejandro M. Armas, Manuel Balparda, Agustina Terenzi, Maria V. Busi, Maria A. Pagani, Diego F. Gomez-Casati

**Affiliations:** 1 Instituto de Biologia Molecular y Celular de Rosario (IBR-CONICET), Universidad Nacional de Rosario, Rosario 2000, Argentina; aarmas@ibr-conicet.gov.ar; 2 Centro de Estudios Fotosintéticos y Bioquímicos (CEFOBI-CONICET), Universidad Nacional de Rosario, Rosario 2000, Argentina; balparda@cefobi-conicet.gov.ar (M.B.); terenzi@cefobi-conicet.gov.ar (A.T.); busi@cefobi-conicet.gov.ar (M.V.B.); pagani@cefobi-conicet.gov.ar (M.A.P.)

**Keywords:** mitochondria, *Arabidopsis*, frataxin

## Abstract

In plants, the cysteine desulfurase (AtNFS1) and frataxin (AtFH) are involved in the formation of Fe-S groups in mitochondria, specifically, in Fe and sulfur loading onto scaffold proteins, and the subsequent formation of the mature Fe-S cluster. We found that the small mitochondrial chaperone, AtISD11, and AtFH are positive regulators for AtNFS1 activity in *Arabidopsis*. Moreover, when the three proteins were incubated together, a stronger attenuation of the Fenton reaction was observed compared to that observed with AtFH alone. Using pull-down assays, we found that these three proteins physically interact, and sequence alignment and docking studies showed that several amino acid residues reported as critical for the interaction of their human homologous are conserved. Our results suggest that AtFH, AtNFS1 and AtISD11 form a multiprotein complex that could be involved in different stages of the iron–sulfur cluster (ISC) pathway in plant mitochondria.

## 1. Introduction

Iron-sulfur (Fe-S) clusters are among the oldest and most versatile cofactors found in nature. Although these clusters can form spontaneously in vitro, because of the high toxicity of Fe^2+^ and S^2-^ the cell needs specialized mechanisms for cluster assembly and insertion into apoproteins [1,2]. Four different types of Fe-S cluster biosynthetic systems have been described: (1) the NIF (nitrogen fixation) system, present in azeotropic bacteria and required for metallocluster biogenesis in nitrogenase [3,4]; (2) the SUF (sulfur mobilization) system, originally identified in *E. coli* and also found in plastids; (3) the ISC (iron–sulfur cluster) system, commonly found in bacteria and mitochondria [5,6]; and (4) the CIA (cytosolic iron-sulfur cluster assembly) system, for cytosolic/nuclear proteins from eukaryotic cells [7]. These systems have several steps in common: first, the acquisition of sulfur from cysteine, catalyzed by a cysteine desulfurase (except for the CIA system); a second stage where the Fe-S groups associated with scaffold proteins begin to be assembled and matured, and a third stage where Fe-S clusters are inserted into an apoprotein [8,9,10,11]. Both plastids and mitochondria can assemble their Fe-S proteins using different machineries that differ in biochemical properties, genetic make-up, and evolutionary origin [8].

The L-cysteine desulfurase is a pyridoxal phosphate-dependent (PLP) homodimeric enzyme that uses L-cysteine as a substrate to produce L-alanine and a protein-bound persulfide [12,13]. In *Arabidopsis*, there are two genes that code for cysteine desulfurases involved in the synthesis of Fe-S groups: AtNFS1, which is located in mitochondria, and AtNFS2 (cpNifS), located in chloroplasts [8,14,15,16,17,18].

Frataxin is a protein that has been widely conserved through evolution in bacteria, yeasts, mammals, and plants without major structural changes [19,20]. Several functions were proposed in which frataxin would be involved, including iron homeostasis and respiration [21], heme metabolism [22], assembly of Fe-S centers [23,24], oxidative phosphorylation and oxidative stress [25], storage of Fe in mitochondria in a water-soluble and non-toxic form [21,26], and recently, its involvement in persulfide transfer [27]. Previously, we described the presence of frataxin from *Arabidopsis* [24,28] and maize [29,30] and the results indicate that it is an essential protein in plants, required for optimal activity of Fe-S proteins and it is also involved in protection against oxidative damage [24,28,31,32,33]. It has been suggested that frataxin would participate in specific steps of the ISC pathway or heme synthesis, where the protein would act as an iron chaperone or donor [9,19,34,35,36], as a positive regulator of cysteine desulfurase in humans [37] or as an activator of the persulfide transfer [26].

In humans, it was described that ISD11 is a small chaperone that binds and stabilizes NFS1 [38,39]. ISD11 is a member of the LYR protein family and it has no ortholog in prokaryotes but is conserved from yeast to humans [40,41]. The depletion of ISD11 causes a high decrease in the levels of some Fe-S proteins in SDH and aconitase activities and the transfer of Fe-S clusters to ferredoxin [38].

It was demonstrated that, within the human mitochondria, frataxin binds the Fe-S biogenesis complex formed by a cysteine desulfurase NFS1, the scaffold protein ISCU, ISD11, and an acyl-carrier protein (ACP) [37,42,43,44] and that the consequences of frataxin deficiency are the same as for the ISD11 deficiency. Thus, it was suggested that ISD11 participate in the early stages of the Fe-S synthesis in humans [38,41,45]; however, there is little information on this protein complex in plants. In previous works, we found that the cysteine desulfurase activity of AtNFS1 was increased in the presence of frataxin (AtFH) [18]. Moreover, we reported that AtNFS1 and AtISD11 regulate the ferrochelatase activity of AtFH in vitro [36].

In this work, we further characterize the AtNFS1 enzyme from *A. thaliana*. Results indicate that this protein interacts with AtFH and AtISD11, forming a multiprotein complex. We also discuss how the interaction between these three proteins would improve the functional efficiency of this ISC complex in the biogenesis of Fe-S clusters in plant mitochondria.

## 2. Results

### 2.1. Effect of Frataxin (AtFH) and AtISD11 on Desulfurase (AtNFS1) Kinetic Parameters

**Figure 1:**
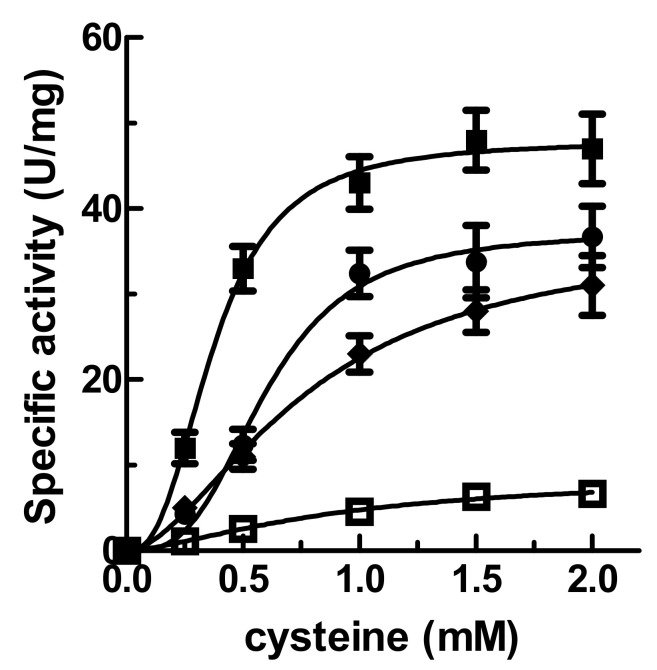
Kinetic assays of AtNFS1:AtISD11:AtFH. L-Ala production from cysteine catalyzed by AtNFS1 in the presence of AtISD11 (1:1, circles); AtFH (1:1, diamonds); AtISD11 and AtFH (1:1:1, black squares) or AtNFS1 alone (empty squares). One unit of activity is defined as the amount of enzyme catalyzing the production of 1 µmol of L-alanine per min. at 37 °C.

It was observed that, in the presence of its substrates, AtNFS1 alone shows low cysteine desulfurase activity (6.20 ± 1.2 U/mg), at least under the conditions studied. However, found that AtNFS1 activity shows a greater activity increase in the presence of AtFH, AtISD11, or AtFH/AtISD11 (1:1:1 molar ratio). The saturation plots are shown in Figure 1. The specific activity of AtNFS1 was measured in the presence of AtISD11 at different substrate concentrations and it displayed a sigmoidal curve with a *V_max_* of 39.40 ± 2.72 U/mg, an *S*_0.5_ of 0.75 ± 0.11 mM, and an *n*_H_ of 2.5 ± 0.5. In the presence of AtFH, a *V_max_* of 32.71 ± 3.26 U/mg, an *S*_0.5_ of 0.67 ± 0.08 mM, and an *n*_H_ of 2.2 ± 0.4 was obtained. However, in the presence of AtFH and AtISD11 (1:1:1 molar ratio), the desulfurase activity showed an increment of about 6-fold in the *V*_max_ (47.83 ± 3.52 U/mg) and an increase of about 3.5-fold in the apparent affinity for cysteine (*S*_0.5_ = 0.23 ± 0.04 mM) respect to the assay using AtNFS1 alone (Figure 1). These results indicate that both, AtISD11 and AtFH would acts as regulators, increasing the desulfurase activity, suggesting that the three proteins could interact forming a multiprotein complex.

### 2.2. Pull-Down Assays and Interaction Studies between AtNFS1, AtFH, and AtISD11

**Figure 2:**
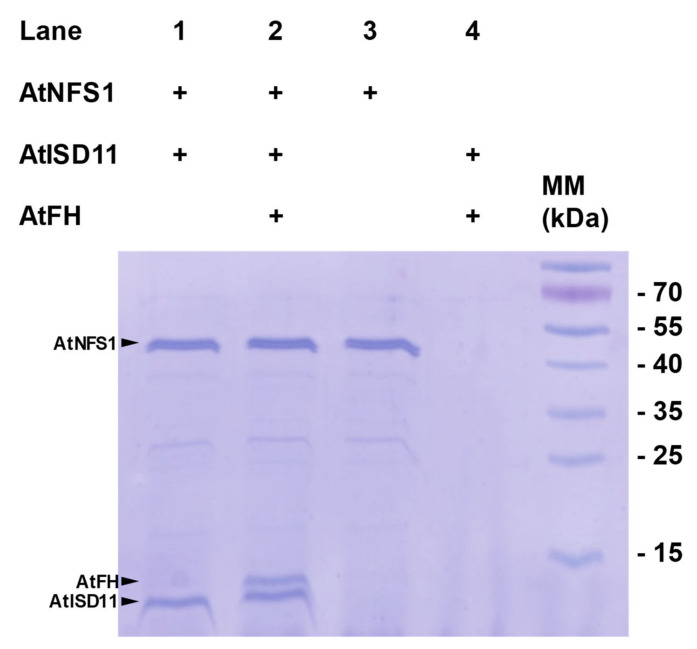
Sodium dodecyl sulfate-polyacrylamide gel electrophoresis (SDS-PAGE) analysis of recombinant proteins. SDS-PAGE analysis of the pull-down assay of the recombinant proteins AtNFS1 (containing a His_6_ sequence), AtISD11 and AtFH. Lane 1, AtISD11 was recovered with AtNFS1; lane 2, AtISD11 and AtFH were recovered together with AtNFS1; lane 3, AtNFS1 recovered from the Ni^+2^ resin; lane 4, non-specific binding control of AtISD11 and AtFH. MM shows the molecular markers used. Arrows indicate the position of each protein band.

To evaluate the possible protein-protein interactions suggested by the kinetic studies, in vitro pull-down assays were performed with purified fractions of AtNFS1-His_6_, AtFH, and AtISD11 (both lacking a His_6_ sequence). After incubating AtNFS1-His_6_ with the Ni^+2^ resin, 50 μg of AtISD11, or 50 μg of AtISD11 and AtFH each, were added and incubated for a further 2 h. After the corresponding washes, resin bound proteins were eluted with 500 μM imidazole and these fractions were analyzed by sodium dodecyl sulfate-polyacrylamide gel electrophoresis (SDS-PAGE). In the presence of AtISD11, we found two protein bands of around 10 kDa (corresponding to AtISD11) and 48 kDa (corresponding to AtNFS1-His_6_), indicating that both proteins physically interact (Figure 2); while in the presence of both, AtFH and AtISD11 it was observed the presence of the 10 kDa and 48 kDa protein bands but also a 14 kDa band, corresponding to AtFH. No protein bands were observed in the SDS-PAGE analysis of the proteins eluted from the resin when AtFH and AtISD11 were incubated in the absence of AtNFS1-His_6_, indicating that both proteins do not bind to Ni^2+^ resin (Figure 2, lane 4). Thus, in agreement with the kinetic experiments, we determined a positive interaction between AtNFS1, AtFH, and AtISD11.

**Figure 3:**
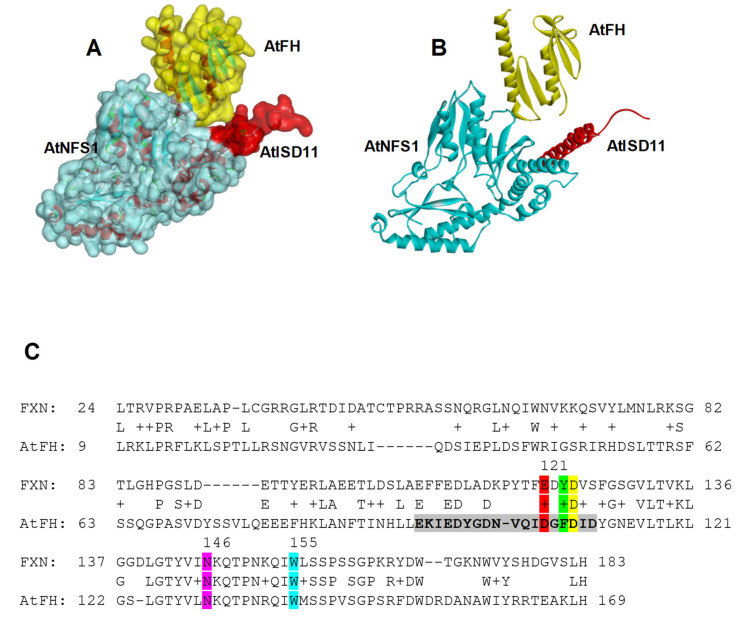

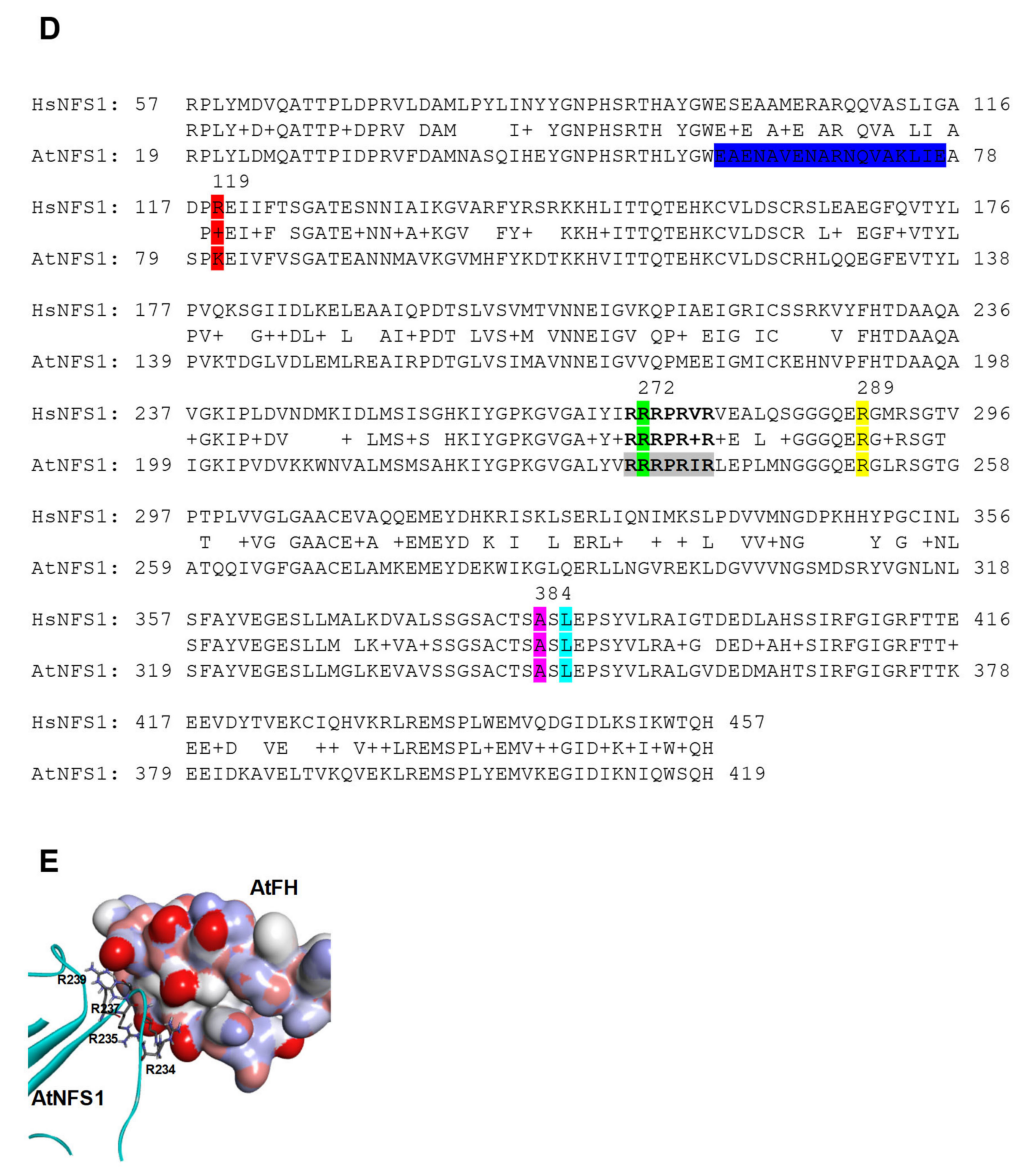
Homology modeling of iron–sulfur cluster (ISC) complex and docking studies. (**A**) Surface and (**B**) ribbon models of the protein complex formed by AtNFS1 (light blue), AtISD11 (red), and AtFH (yellow). The docking model was predicted using HADDOCK 2.4. (**C**) Amino acid sequence alignment of human (FXN) and *Arabidopsis* frataxin (AtFH) and (**D**) human (HsNFS1) and *Arabidopsis* (AtNFS1) cysteine desulfurases. The amino acids highlighted with color in (**C**) interact with the amino acid with the same color in (**D**). The amino acids marked in grey in (**C**) are part of the negatively charged patch in AtFH. Amino acids marked in grey in D are part of the Arg-rich patch in AtNFS1, and those marked in blue are part of the negatively charged helix region that interacts with the α2-helix from AtISD11. (**E**) Interaction of the acidic AtFH patch with Arg residues on AtNFS1.

The interaction between AtNFS1, AtFH, and AtISD11 was studied in greater depth by protein alignment and docking studies. The studies carried out by Haddock 2.4 [46] indicated that the most energy-efficient model is the one showed in Figure 3A,B, suggesting an interaction between AtFH–AtNFS1 and AtISD11–AtNFS1.

Amino acid residues involved in both interactions have been identified by the alignment of each protein sequence with the sequences from the homologous proteins present in humans. As mentioned previously, it was found that NFS1 and ISD11 interact in human mitochondria, suggesting that the last would be required for the assembly of Fe-S proteins [38]. Moreover, the critical amino acid residues for the interaction have been mapped [47]. Recently, it was reported the structure of a heterodecamer multiprotein complex that would be key for the biosynthesis of mitochondrial Fe-S clusters in humans containing two copies of each of NFS1, frataxin (FXN), ISCU, ISD11, and ACP [48].

It has been reported that a positively charged Arg-rich patch (Arg271-Arg277, RRRPRVR) in NFS1 is critical for the interaction with acidic residues that form a negative patch in FXN (loops α1, loop α1-β1, and the start of β1). Also, FXN Asp124 form a salt-bridge with NFS1 Arg289, and carbonyl backbone groups from FXN residues Glu121 and Tyr123 could interact via hydrogen-bonds with NFS1 Arg119 and Arg272. It was also reported that FXN directly interacts with a second NFS1 subunit near the catalytic site via hydrophobic interaction between FXN Trp155 and NFS1 Leu386, and a hydrogen-bond between FXN Asn146 and NFS1 Ala384 [48].

Thus, after protein sequence alignment we found high conservation of the residues involved in AtFH-AtNFS1 binding. Glu121 is replaced by an Asp106 and Tyr123 align with a Phe108 in AtFH, while the Asp124 is conserved (Asp109 in AtFH). In addition, Asn146 and Trp155 are also conserved in AtFH (Asn130 and Trp139, respectively) (Figure 3C). The amino acid residues shown in color in Figure 3C,D would be those involved in the interaction between AtFH and AtNFS1 (the residues of the same color are predicted to interact with each other). On the other hand, we found that Arg119 from NFS1 is replaced by a Lys81, and Arg272, Arg289, Ala384, and Leu386 are conserved in AtNFS1 (Figure 3D). Furthermore, we found that the Arg-rich patch found in human NFS1 is also well conserved in AtNFS1 (Arg233-239, RRRPRIR) (see Figure 3D). As mentioned above, this positively charged patch would be critical for the interaction with the acidic patch (Glu94-D111) present in AtFH (Figure 3E).

**Figure 4:**
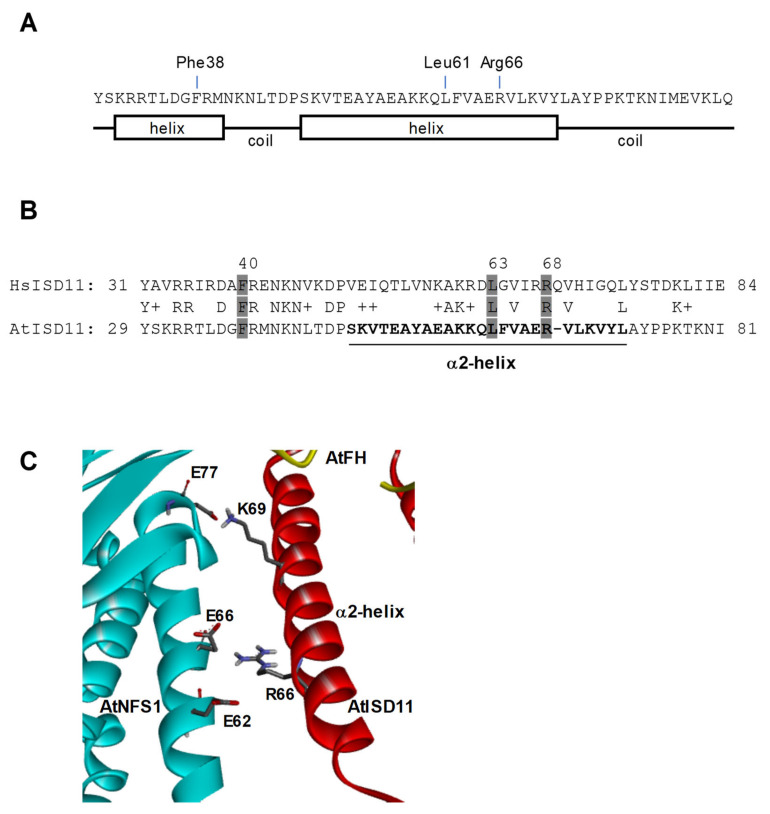
Secondary structure, sequence alignment and docking studies of AtISD11. (**A**) Secondary structure of AtISD11 showing the amino acids which interact with AtNFS1 in the protein complex. (**B**) Amino acid sequence alignment of human (HsISD11) and *Arabidopsis* (AtISD11) ISD11. Amino acids in grey are predicted to be critical for HsISD11 function. The region marked in bold, from Ser48 to Leu72 in AtISD11 is the second helix region (α2-helix) which interacts with AtNFS1. (**C**) Modeled interaction between AtNFS1 (light blue) and AtISD11 (red) showing important residues possibly involved in the interaction.

The structural model obtained for the AtFH-AtNFS1-AtISD11 complex also shows an interaction between AtNFS1 and AtISD11. A secondary structure prediction shows that AtISD11 has 2 helix domains close to the N-terminus of the protein and they are well conserved (Figure 4A). It was described that Arg68 from human ISD11 is critical for the interaction with NFS1, but also other amino acid residues are involved in ISD11 stabilization of NFS1 such as Phe40 and Leu63 [47]. We found that Phe40, Leu63, and Arg68 are conserved in AtISD11 (Phe38, Leu61, and Arg66, AtISD11 numbering) (Figure 4B). Interestingly, Leu61 and Arg66 are part of the second helix region of AtISD11 (Ser48-Leu72) that is predicted to interact with AtNFS1. It is possible that the conserved Arg66 form a salt bridge with Glu62 and/or Glu66 present in the N-terminal region of AtNFS1, and the Lys69, also present in the second helix of AtISD11, would also interact with a Glu77, which might help to stabilize the enzyme (Figure 4C). It is interesting to note that other residues present in the C-terminal region and reported being involved in ISD11 function are not conserved in AtISD11. Furthermore, in general, there is no conservation at C-terminal ends of both proteins, suggesting a different role of this region for the plant protein. The analysis of the predicted model and the identification of key amino acids present in AtFH, AtNFS1, and AtISD11 showed that some structural characteristics are conserved with respect to the human ISC complex, however, some differences were found. It was reported that HsFH can interact with ISD11 [42], and also with ISCU at the interface that this protein forms with Nfs1 [48]. We were unable to find any interaction between AtFH and AtISD11 or AtFH and AtISU1 (not shown), suggesting that AtISU1 would have weak binding or would not bind to the proposed plant ISC complex.

### 2.3. Evaluation of the Attenuation of Fenton Reaction in the Presence of AtFH, AtISD11, and AtNFS1

**Figure 5:**
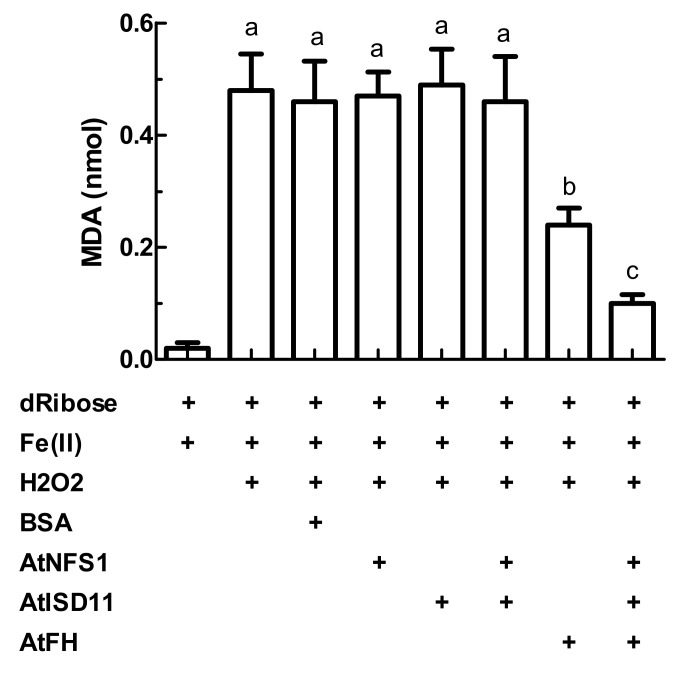
**Oxidative degradation assays.** Production of malondialdehyde from dRibose, Fe(II), and H_2_O_2_ in the absence or presence of the recombinant proteins AtFH, AtNFS1, and AtISD11 or bovine serum albumin (BSA). Letters indicate significant differences (*p* < 0.05).

Previously, it was reported that frataxin could act as an iron chaperone protein [31,49], and in this way, its presence could attenuate oxidative damage by metals. Since AtFH forms a complex with AtNFS1 and AtISD11, we decided to perform the attenuation test of the Fenton reaction in the presence of the multiprotein complex (Figure 5). The AtFH concentration used in this experiment is in excess respect to Fe (2:1 molar ratio). It is important to note that previously it has been analyzed only the ability to attenuate Fenton reaction by frataxins from different organisms alone and not in the presence of other proteins [29,31].

Controls in the presence of dRibose, Fe(II) and H_2_O_2_ and bovine serum albumin (BSA) show that in the absence of AtFH, about 0.5 nmol of malondialdehyde were obtained. The incubation of AtNFS1 and/or AtISD11 in the absence of AtFH did not significantly affect also the levels of malondialdehyde generated (Figure 5). However, in the presence of AtFH, a decrease of about 40% in the production of malondialdehyde was observed, suggesting both a decrease of the amounts of free radicals produced, and that only AtFH is able to sequester Fe^2+^ (and/or Fe^3+^), Figure 5. Furthermore, after the incubation of the three proteins, AtFH, AtNFS1, and AtISD11 in the reaction media, a decrease in the amount of malondialdehyde produced was observed of about 80%, approximately 2-fold more than the decrease observed in the presence of AtFH alone. These facts reinforce the hypothesis that these three proteins interact forming a multiprotein complex and that this complex would be more efficient in carrying out different stages of the synthesis of Fe-S groups.

## 3. Discussion

In this study, we characterized the structure and possible functions of the cysteine desulfurase AtNFS1, frataxin (AtFH), and the ISD11 protein (AtISD11) from *Arabidopsis thaliana*. Although there are previous works about the characterization of these proteins in other organisms such as bacteria, yeasts, plants, and humans, there is little information about their role in the ISC pathway of synthesis of Fe-S groups in photosynthetic organisms [19,21,33,34,37,38,42,45,50,51].

It was previously described that human NFS1 interacts with ISD11 and this interaction stabilizes NFS1, preventing its aggregation [38,45,47]. The amino acid residues involved in such interaction were mapped and results showed that some residues present in the helix regions of the ISD11 protein would have an essential role in the interaction [47]. Moreover, it has recently been shown in human mitochondria that the complex involved in the synthesis of Fe-S clusters is a heterodecamer composed of five proteins: NFS1, frataxin, the scaffold protein ISCU, ISD11, and an acyl-carrier protein (ACP) [48].

After the assay of the enzymatic activity and docking studies, we proposed that AtNFS1 interacts with AtFH and AtISD11. This interaction was confirmed by pull-down assays using the recombinant proteins. Our data agree with the formation of the human ISC complex mentioned before [48]. Although some regions were omitted in the docking simulation possibly because they could be disordered or have high mobility, such as the Asn245-Ser255 region in AtNFS1, we found that the three proteins show almost full conservation of the residues involved AtNFS1-AtFH and AtNFS1-AtISD11 interactions.

It has been reported that the core structure of frataxin is highly conserved among diverse organisms. Frataxin has a unique fold composed of two terminal α-helices and five to seven antiparallel β-strands forming a compact α/β sandwich [51,52]. Although there are differences in sequence and length, the acidic residues located in the α1-helix, β1-strand, and the α1-β1 loop regions are highly conserved. These acidic residues form a negatively charged surface that covers about a quarter of frataxin’s total accessible surface and it has been suggested that they could participate in metal binding and/or protein–protein interactions [52]. In our docking model, we found that the negative patch located in the α1/β1/α1-β1loop of AtFH (Glu94-D111) could interact with the conserved RRRPRIR positive patch in AtNFS1 (Arg233-Arg239). This data is consistent with the characteristics of the interaction NFS1-FH described for the ISC complex from humans [48].

Our model also predicts that AtNFS1 interacts with AtISD11. Comparison of amino acid sequences and homology modeling showed that AtISD11 has a high similarity to the human homolog only in the N-terminal region of the protein. The C-terminal region is less conserved, suggesting a different function in the different lineages. However, we found some residues present in AtISD11 helices 1 and 2 that could be involved in the interaction with AtNFS1, especially Arg66 (Arg68 in ISD11). The structural model also shows that this residue could interact with a negatively charged helix alpha segment of AtNFS1.

On the other hand, our data showed that AtISD11 and AtFH are positive regulators for AtNFS1 activity. Here, we report that AtNFS1 has a specific activity of 39.4 U/mg in the presence of AtISD11, which is increased by AtFH. Interestingly, the kinetic analysis of AtNFS1 in the presence of AtISD11 showed a hysteretic behavior, with a lag phase of several minutes (not shown). It is known that hysteresis has physiological importance to buffer radical changes in the concentration of metabolites in biological systems [53]. The interaction between AtNFS1 and AtISD11 would be crucial since in the absence of the latter it was observed that AtNFS1 shows very low cysteine desulfurase activity, as was recently reported in humans [27,39]. Moreover, AtFH directly stimulates the cysteine desulfurase activity. This behavior was already reported in many organisms such as mammals, yeast, and plants [18,54,55,56,57]. It was suggested that frataxin would act as an allosteric switch that activates the ISC Fe-S cluster biosynthetic complex. For example, in yeasts, frataxin directly modulates the NFS1 activity by exposing its substrate-binding sites [55]. Thus, it is possible to postulate that AtNFS1 would exist in two different conformations, a low activity one when AtFH is absent, and a second form that exhibits higher activity, triggered by the binding of AtISD11 and AtFH. The interaction of both proteins with AtNFS1 could be a unique regulatory mechanism in which the lack of binding of AtFH prevents premature activation of AtNFS1, and the AtFH binding would be required in specific cellular conditions, such as elevated O_2_ pressure [58]

It was reported that frataxin is also involved in Fe-related steps of Fe-S and heme synthesis in mitochondria [8,9,34,59,60]. It cannot be denied that the absence or decrease of frataxin levels cause numerous alterations in iron metabolism in different organisms. However, there is controversy about the specific molecular function/s that frataxin would perform in relation to Fe metabolism. We have previously demonstrated that AtFH was able to bind and maintain Fe^2+^ in solution under atmospheric O_2_ pressure [36], as was reported for the human homolog [61], and that AtFH has in vitro ferrochelatase activity. It is interesting to note that the recent proposed ferrochelatase activity of AtFH is regulated by the formation of the proposed AtFH-AtNFS1-AtISD11 complex [36]. This regulation of AtFH activity was also observed in the oxidative degradation assays carried out in this work in which the attenuation of the Fenton reaction was more efficient in the presence of the complex formed by the three proteins. Although the chemistry behind the attenuation of the Fenton reaction by frataxins, and its relation with Fe metabolism, are not clear, this activity has been described as characteristic of yeast frataxin [29,31,49]. Also, the Fenton reaction is related to the presence and generation of reactive oxygen species, which occurs mainly in aerobic conditions. Recently it was demonstrated that frataxin null yeast, human cells, and nematodes are fully viable in ambient with 1% oxygen (hypoxic conditions) [58]. Frataxin is required to sustain the Fe–S cluster synthesis rate under environmental oxygen pressure. Additionally, decreased levels of frataxin triggers Fe deficiency responses and Fe accumulation in different organisms like yeast [21,62], flies [63], *Arabidopsis* [32,64], and humans [65,66], although it is not clear if this is a primary or secondary event. Indeed, there is a link between iron, oxygen, and frataxin, and the increased attenuation of the Fenton reaction by the AtFH-AtNFS1-AtISD11 complex is suggestive of the biological relevance of the reaction, and its relation with some of the molecular function of frataxin.

In conclusion, our results suggest that AtFH, AtNFS1 and AtISD11 form a multiprotein complex in *Arabidopsis* mitochondria. We found that AtFH and AtISD11 modulate the desulfurase activity of AtNFS1, indicating that the complex would have an important role in the early stages of Fe-S cluster synthesis in plant mitochondria. Moreover, the complex formed by these three proteins could be important in mitigating oxidative damage in plant mitochondria. In support of the data presented here, we recently reported a decrease of AtISD11 and AtFH mRNA in AtNFS1 deficient plants, while these transcript levels were increased when AtNFS1 was overexpressed, indicating the existence of a regulatory link between these genes [67]. We have also reported that the altered levels of AtNFS1 and AtFH affect the iron and sulfur homeostasis in plants [67] and we suggested a model in which both metabolisms are regulated by an integrated signal triggered by the Fe-S biosynthetic pathway. The mutual regulation of AtFH and AtNFS1 (and AtISD11) could be related to the nutritional requirements of Fe and S in mitochondria, and the formation of a functional AtFH-AtNFS1-AtISD11 complex as a part of the ISC Fe–S biosynthetic pathway, would be important for the regulation of the iron and sulfur homeostasis in plants.

## 4. Materials and Methods

### 4.1. Cloning, Expression and Purification of AtNFS1, AtFH, and AtISD11

AtFH (NP_192233) was cloned as previously described [31], while AtNFS1 (NP_201373) and AtISD11 (NP_200930) were obtained by gene synthesis (Genscript) with codon optimization for heterologous expression in *E. coli* cells as described [36]. Briefly, the sequence coding for the mature form of AtFH was cloned in pET24a vector with a stop codon preventing the transcription of the His_6_ tag in its C-terminal region; mature AtNFS1 coding sequence was cloned into a pET32a plasmid containing a His_6_ tag and a TEV protease cleavage site and the mature AtISD11 CDS sequence was cloned into a pRSFDuet-1 plasmid, also containing a His_6_ tag and a Tobacco Etch Virus (TEV) cleavage site. Protein expression and purification were performed as described previously [31,36].

### 4.2. Cysteine Desulfurase Assay

The cysteine desulfurase activity was measured in a reaction mixture containing 25 mM Tris-HCl, pH 8, 100 mM NaCl, 10 μM PLP, 100 μM DTT, and different concentrations of L-cysteine (0–2 mM), as indicated in each case. The assays were initiated by the addition of the recombinant enzyme AtNFS1 and incubated at 37 °C for different time intervals (0–180 min). To determine the conditions in which the steady-state is reached, different concentrations of recombinant AtNFS1 were tested. The production of L-alanine from L-cysteine was quantified by the colorimetric method of ninhydrin with modifications [68,69]. Briefly, after the reaction, a volume of ninhydrin in ethanol (0.2% *W/V*) was added to the mixtures, incubated at 90 °C for 10 min, centrifuged at maximum speed for 10 min and the supernatants were kept on ice until the spectrophotometric measurement at 565 nm. The complex formed by L-cysteine and ninhydrin did not show a significant absorbance at 565 nm according to that reported in the literature [68]. The concentration of L-alanine was estimated from a standard curve made with different amounts of L-alanine (0 to 2 mM), obtained under the same conditions as those described for the reactions. One unit of activity was defined as the amount of enzyme that catalyzes the production of 1 nmol of L-alanine per minute at 37 °C. Kinetic parameters, *S*_0.5_ (concentration of substrate that gives half of the maximal velocity), *n*_H_ (Hill number), and the *V*_max_ (Maximal velocity), were determined using different concentrations of the substrate (L-cysteine) and they were calculated using the steady-state velocities (*V*_ss_) [70]. To determine how the cysteine desulfurase activity of AtNFS1 is affected when the protein interacts with other enzymes of the pathway such as AtISD11 and AtFH, we proceeded in the same manner described above with the addition of different amounts of those enzymes.

### 4.3. Pull-Down Assays

Pull-down assays were carried out as described previously [18]. Briefly, 20 µg of purified His_6_-tagged AtNFS1 was bound to a Ni^2+^-Sepharose high-performance resin (GE Health-care Bio-Sciences) previously equilibrated with binding buffer (20 mM NaH_2_PO_4_, pH 7.4, 50 mM NaCl, 1 mM 2-mercaptoethanol, and 20 mM imidazole), followed by incubation with 50 μg of AtFH and/or 50 μg of AtISD11, both proteins without the His_6_ tag (removed using TEV protease). After 2 h of incubation with shaking at 4 °C, three washes with 5 mL of binding buffer were carried out and the retained proteins were eluted from the resin by the addition of 500 mM imidazole. The results were analyzed by 15% (*W/V*) SDS-PAGE.

### 4.4. Oxidative Degradation Assays

To assess the ability to attenuate the Fenton reaction, the inhibition of malondialdehyde production was measured after the addition of thiobarbituric acid as previously described [31,49]. Briefly, a mixture of 15 µM Fe(II), 8 µM H_2_O_2_ and 5 mM of 2-deoxyribose (Fluka) were incubated in 10 mM Hepes–KOH, pH 7.0 in the absence or the presence of recombinant proteins (30 µM of AtFH, AtNFS1, and/or AtISD11). The final volume was 100 µL. After 30 min at 25 °C, 4% (*V/V*) phosphoric acid, and 1% (*W/V*) thiobarbituric acid were added to the reaction mixture (100 µL each). The samples were incubated for 10 min at 100 °C and after cooling on ice, 75 µL of 10% SDS was added to each tube. The amount of malondialdehyde was determined spectrophotometrically, by measuring absorbance at 532 nm.

### 4.5. Additional Methods

SDS-PAGE was performed using 12% (*W/V*) gels as described by Laemmli [71] and developed by Coomassie blue staining. Total protein concentration was determined as described by Bradford [72]. The 3-D models of the protein structures were obtained from @TOME v2.3 platform [73]. The AtISD11 model was made using the human ISD11 as a template (PDB: 5WGB) and analyzed using ProSA-web [74]. Docking predictions were made using HADDOCK 2.4 with default settings and using active residues reported to be involved in binding in the human ISC complex (see Figure 3 and Figure 4) [46]. The 3-D visualization was done using PyMOL (PyMOL by Schrödinger, [75]). The alignment of protein sequences was performed by using the ClustalW algorithm from Unipro UGENE v.1.10.4 program with default parameters [76].

### 4.6. Statistical Analyses

The significance of differences was determined using one-way analysis of variance (ANOVA) and Tukey’s test. Values statistically different (*p* < 0.05) are denoted with different letters.
